# Perceived knowledge and attitudes toward fall prevention among nurses and healthcare assistants: a Cross-Sectional survey study

**DOI:** 10.1080/07853890.2025.2559127

**Published:** 2025-09-19

**Authors:** Claudio Tana, Alessandro Di Risio, Samanta Moffa, Constantino Gallo, Raffaele di Nardo, Mauro Palmieri, Francesco Cipollone, Delia Racciatti

**Affiliations:** ^a^ Geriatrics Clinic, SS. Annunziata Hospital of Chieti, ASL Lanciano Vasto, Chieti, Italy; ^b^ Risk Management Unit, SS. Annunziata Hospital of Chieti, ASL Lanciano Vasto, Chieti, Italy; ^c^ Department of Pharmacy, “G. d’Annunzio” University of Chieti-Pescara, Chieti, Italy; ^d^ Research and Innovation Unit, SS. Annunziata Hospital of Chieti, ASL Lanciano Vasto, Chieti, Italy; ^e^ Executive Healthcare Management Unit, SS. Annunziata Hospital of Chieti, ASL Lanciano Vasto, Chieti, Italy; ^f^ Internal Medicine Unit, G’Annunzio University of Chieti-Pescara, Chieti, Italy

**Keywords:** Falls, prevention, risk management, nursing education, healthcare assistants, patient safety

## Abstract

**Background:**

Falls are among the most common adverse events in hospitals, with major clinical and legal consequences. Despite existing prevention guidelines, fall-related incidents remain frequent, underscoring the need to evaluate healthcare workers’ awareness and adherence to institutional protocols.

**Aim of the study:**

To assess perceived knowledge, attitudes, and barriers to fall prevention among nurses and healthcare assistants, in order to identify gaps and inform targeted strategies.

**Methods:**

A cross-sectional survey was conducted among 295 nurses and healthcare assistants across multiple departments of the ASL Lanciano Vasto Chieti, Italy. The self-administered questionnaire collected demographic data, knowledge, attitudes, perceived barriers, and awareness of institutional protocols. Data were analyzed using descriptive statistics, chi-square tests, ANOVA, and logistic regression (p < 0.05).

**Results:**

Greater professional experience was significantly associated with participation in fall prevention training (*p *< 0.005). While most participants reported using fall risk assessment scales, uncertainties persisted regarding their limitations. Frequently cited barriers included limited availability of equipment and underreporting of incidents, often linked to incomplete procedural knowledge and poor dissemination of educational materials (*p *< 0.0001). Participation in training correlated with higher adherence to protocols and improved fall risk assessment practices (*p* < 0.0001).

**Conclusions:**

Overall awareness of fall prevention was adequate, but variability in training, incident reporting, and resource access remain critical gaps. Reinforcing educational programs, ensuring consistent dissemination of materials, and fostering evidence-based, individualized strategies could enhance adherence to best practices and reduce fall-related events.

## Introduction

Patient falls are a significant concern in hospital settings, representing one of the most common adverse events affecting patient safety. Falls can lead to severe complications, including fractures, head trauma, prolonged hospitalization, and increased healthcare costs. Given the aging population and the complexity of hospitalized patients, fall prevention has become a priority for healthcare systems worldwide [1].

Nurses and healthcare assistants play a pivotal role in fall prevention as they are the frontline healthcare providers responsible for patient monitoring, risk assessment, and intervention implementation. Effective fall prevention strategies include environmental modifications, patient education, risk assessment tools, and the use of assistive devices. While risk assessment tools are widely used in clinical settings, recent international guidelines have questioned their predictive accuracy and effectiveness in preventing falls. Therefore, skepticism among nursing staff toward these tools may reflect an evidence-aligned perspective rather than a knowledge gap [2].

A significant reduction in falls can be achieved through a multi-level integrated program, including education, rehabilitation, organizational and environmental modifications, and nutritional support [3].

Early identification of the main risk factors for in-hospital falls, such as the use of antidepressants, sedatives, and antipsychotics, as well as a previous history of falls, can help tailor preventive measures for high-risk patients [3]. These pharmacological and clinical vulnerabilities are often intertwined with broader geriatric syndromes, including frailty and sarcopenia, that not only predispose older adults to functional decline but also significantly increase the risk of in-hospital falls. Emerging evidence suggests that these syndromes are influenced by gut microbiota alterations, further highlighting the complex interplay between biological, pharmacological, and functional factors in fall risk among older patients [4,5].

Several studies have highlighted the importance of nurses and healthcare assistants’ awareness and perceived knowledge in reducing fall incidents. However, varying perspectives on the utility of fall risk assessment tools persist, which may be attributable not to misunderstanding, but to critical engagement with the literature and clinical experience. Aligning staff training with current evidence, which emphasizes multifactorial and team-based approaches over tool-based stratification, is therefore essential. Understanding these factors can provide valuable insights into improving training programs, developing evidence-based interventions, and fostering a culture of patient safety [3].

Nursing staff play a crucial role not only in the ongoing monitoring of clinical parameters, but also in the prevention of adverse events, such as falls, which, in a hospital setting, can significantly affect the clinical course and treatment outcomes [3].

This study aims to assess the perceived knowledge and attitudes of hospital nurses and healthcare assistants regarding fall prevention, identifying potential gaps and areas for improvement. By analyzing nurses and healthcare assistants’ self-reported awareness and beliefs, we aim to provide recommendations for targeted educational programs and policy enhancements to optimize patient safety and reduce fall-related complications in hospital settings.

## Materials and methods

### Study design and setting

This study employed a cross-sectional survey design to assess nurses and healthcare assistants’ perceived knowledge and attitudes toward fall prevention in hospital settings. Participants were invited *via* institutional email to take part in the study and completed the questionnaire through an online form (Supplementary material: survey.pdf). The questionnaire was designed by the authors based on a review of the literature and existing clinical guidelines related to fall prevention [1–3]. All questions were close-ended, using either multiple-choice or Likert-scale formats, and all responses were collected electronically. No paper-based surveys were administered. The survey was conducted in multiple hospital departments of the ASL2 Lanciano Vasto Chieti, located in Abruzzo, Italy, including medical, surgical, and emergency units, to ensure a comprehensive representation of nursing staff. The ASL2 Lanciano Vasto Chieti is a public local health authority that serves a population of approximately 390,000 residents across the province of Chieti. It comprises a network of hospitals and territorial healthcare services, including the SS. Annunziata General Hospital of Chieti, which functions as the main hub hospital, as well as peripheral facilities such as the Renzetti Hospital in Lanciano, the San Pio Hospital in Vasto, and smaller district hospitals. This multisite structure enabled the inclusion of a broad and representative sample of nursing staff working in diverse clinical environments, thus enhancing the generalizability of the findings within similar regional healthcare contexts.

### Participants

Registered nurses and healthcare assistants working in hospital settings were invited to participate in the study. Inclusion criteria included active employment in patient care roles, at least six months of work experience, and willingness to complete the survey. Nurses and healthcare assistants from specialized units (e.g. pediatric, maternity) were excluded to maintain focus on adult patient care.

### Data collection

Data were collected through an anonymous, self-administered questionnaire distributed electronically. The questionnaire consisted of three main sections:

1. Demographics: Age, gender, years of experience, and department.
2. Knowledge Assessment: Questions evaluating familiarity with fall risk factors, prevention strategies, and adherence to safety protocols.
3. Attitudes and Perceived Barriers: Assessment of confidence in implementing fall prevention measures, perceived challenges, and institutional support.

### Statistical analysis

Descriptive statistics (frequencies, percentages) were used to summarize participant characteristics and survey responses. Chi-square tests were applied to examine associations between experience levels and knowledge gaps. Logistic regression was performed to identify factors influencing adherence to fall prevention protocols. Statistical significance was set at *p* < 0.05. All analyses were conducted using GraphPad Prism (GraphPad software, Inc., Boston, MA, USA). Univariate analysis was performed using the *t*-test and ANOVA. Correlation analysis was performed based on the questionnaire responses of nurses and healthcare assistants.

### Ethical approval and participation statement

This study was conducted in accordance with the principles of the Declaration of Helsinki. The research involved an anonymous, voluntary survey among healthcare professionals and did not collect any personal, sensitive, or health-related data. The study protocol was reviewed and approved by the Healthcare Management Unit of ASL2 Lanciano Vasto Chieti. Participants were clearly informed, prior to submitting their responses, that the survey was being conducted exclusively for research and statistical purposes, and their voluntary submission of the questionnaire was an informed act of participation. In accordance with the provisions of the Italian Data Protection Code (Legislative Decree No. 196/2003, as amended by Legislative Decree No. 101/2018) and the General Authorization No. 9/2016 issued by the Italian Data Protection Authority, no formal consent was required for the use of fully anonymized data. For the same reason ethics committee approval was not required, as the study did not involve any clinical intervention nor the processing of any personal, sensitive, or health-related data.

## Results

The survey gathered responses from 295 nurses and healthcare assistants, providing valuable insights into their perceived knowledge and attitudes toward fall prevention in hospital settings. Table 1 shows the main results of the survey conducted among nurses and healthcare assistants at ASL2 Lanciano Vasto Chieti regarding their perceived knowledge and attitudes toward fall prevention.

**Table 1.** Summary of survey results on the perceived knowledge and attitudes toward fall prevention among nurses and healthcare assistants at ASL2 Lanciano Vasto Chieti.

	Nurse and healthcare assistants	Percentage
1. Years of experience		
-less than 5 years	54	18.30%
-between 5 and 10 years	52	17.63%
-more than 10 years of experience	189	64.07%
2. Fall risk assessment scales identify patients who are more likely to fall because they have one or more physiological problems.		
Yes	259	12.20%
No	36	87.80%
3. Do you believe the company where you work has sufficient technological requirements for managing falls? (Electric beds, safety rails)		
Partially	131	44.40%
Yes	74	25.08%
No	90	30.50%
4. Can patient falls be prevented by providing them with as safe an environment as possible? (For example: a well-lit path to the bathroom, a room free of clutter)		
True	284	96.30%
False	11	3.70%
5. When there is effective communication between nurses and healthcare workers, is it likely that patients themselves will follow the falls prevention plan?		
True	262	88.80%
False	33	11.20%
6. Patients classified as low risk for falling do not require a falls prevention assessment.		
True	20	7.00%
Fase	275	93.00%
7. If you were to find yourself in the situation of having to report an accidental fall, would you feel uncomfortable and/or intimidated in doing so?		
Yes	77	26.20%
No	218	73.80%
8. Are you familiar with our company’s current procedure for “Prevention and hospital management of patient falls”?		
Yes	226	76.61%
No	69	23.38%
9. Is the “Fall Risk” information brochure for patients and family members still available for delivery to your hospital?		
Yes	154	52.20%
No	141	47.79%
10. Have you ever attended training courses and/or seminars on falls in hospital?		
Yes	106	35.90%
No	189	64.10%
11. Would you like to participate in training courses that address the phenomenon of falls in hospitals?		
Yes	273	92.55%
No	22	7.45%
12. Do you believe the company where you work has sufficient technological requirements for managing falls? (Electric beds, safety rails)		
Yes	74	25.08%
No	90	30.51%
Partially	131	44.41%
13. Have you ever reported or helped report an accidental patient fall to the Quality and Clinical Risk Office?		
Yes	127	43.05%
No	168	56.95%

### 1. Demographics and work experience

**Figure 1.**
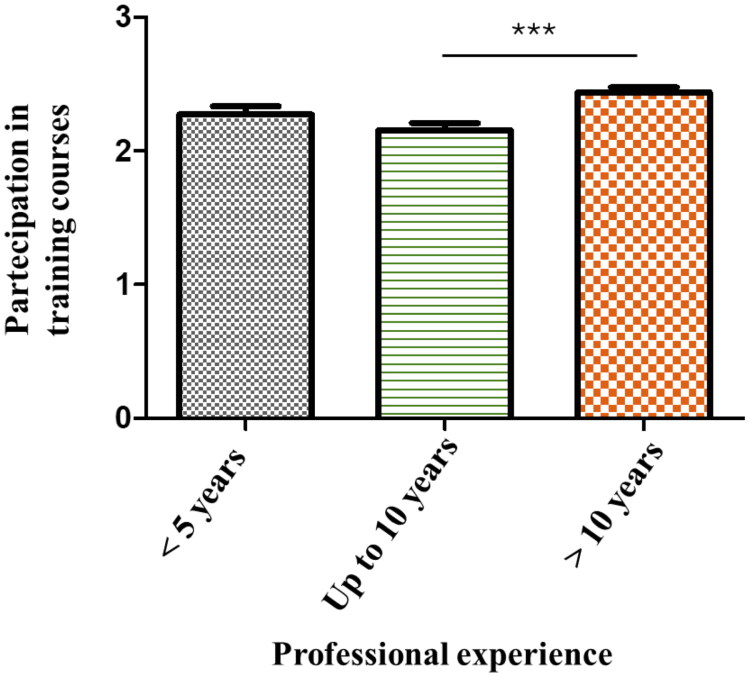
The histogram shows the statistical correlation between professional experience (categorized as less than 5 years, between 5 and 10 years, and more than 10 years) and the participation in training courses on fall prevention.

The majority of participants had significant clinical experience, with 64.1% of nurses and healthcare assistants reporting more than 10 years of experience, while 17.6% had between 5 and 10 years, and 18.3% had less than 5 years. In our study, greater professional experience was significantly associated with higher participation in hospital-based fall prevention training courses (*x*^2^ = 8.19, *p* value < 0.005, Figure 1).

Regarding workplace distribution, most nurses and healthcare assistants worked in medical and surgical units, with smaller representations from emergency and intensive care settings. The Cardiology and Coronary Care Units (CCU) had the highest number of respondents (24 nurses and healthcare assistants).

### 2. Knowledge of fall prevention strategies

When assessing knowledge of fall risk factors, 87.8% of respondents recognized that risk assessment scales help identify patients at higher risk of falling due to physiological conditions. However, 12.2% of nurses and healthcare assistants (36 respondents) indicated that these scales were not predictive.

In terms of environmental modifications, 96.3% of nurses and healthcare assistants agreed that ensuring a safe hospital environment, such as proper lighting, removing clutter, and using appropriate footwear, plays a crucial role in fall prevention. A small percentage (3.7%) disagreed.

Furthermore, 44.4% of respondents indicated that their healthcare facility partially meets the technological requirements for effective fall risk management, such as the availability of electric beds. 25.1% believed that the facility does not meet these requirements, while 30.5% felt that the necessary technologies are adequately available.

Interprofessional communication was also considered essential, with 88.8% of nurses and healthcare assistants stating that collaboration between nurses and healthcare assistants improves adherence to fall prevention protocols.

### 3. Attitudes toward fall prevention measures

Regarding the use of technological interventions, 93% of nurses and healthcare assistants supported activating bed and chair alarms for all high-risk patients, if available. However, 7% expressed skepticism, likely due to concerns about overuse, alarm fatigue, or potential distress to patients.

### 4. Barriers to fall prevention

- Lack of Equipment (45.6%): Nearly half of the nurses reported that hospitals lack essential fall prevention equipment such as electric beds, protective side rails, and fall alarm systems.
- Reporting Hesitancy (26.2%): A significant number of nurses expressed hesitancy in reporting patient falls, suggesting cultural barriers to incident reporting.

**Figure 2.**
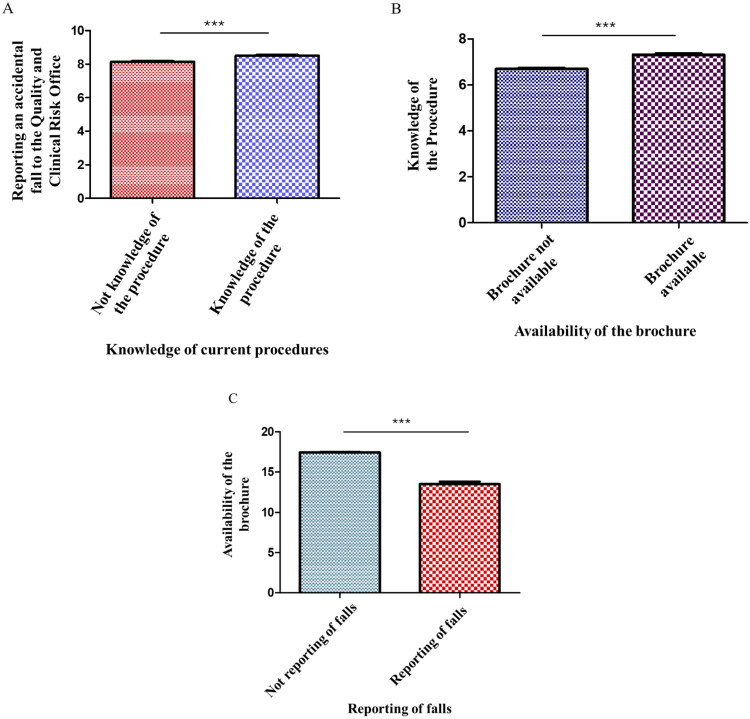
A) The histogram shows the statistical significant correlation between knowledge of current procedures and the probability of reporting an accidental fall to the Quality and clinical risk Office; B) the histogram shows the statistical significant correlation between the availability of the brochure and the knowledge of the procedure in force in our company; C) the histogram shows the statistical significant correlation between the absence or presence of brochures and the reporting of falls.

Familiarity with company protocols, specifically the procedure entitled “Prevention and Hospital Management of Patient Falls”, appears to be directly associated with the likelihood that nurses and healthcare assistants will report an incident. Statistical analysis revealed that staff members who are knowledgeable about the current reporting procedures are significantly more likely to notify the Quality and Clinical Risk Office in the event of a patient fall, as illustrated in Figure 2A (*p* < 0.0001).

On the other hand, the “Fall Risk” informational brochure, intended for patients and family members, is not consistently available across all hospital units. This limited distribution contributes to insufficient awareness of preventive procedures and may foster a perception of inadequate information, which in turn can lead to the underestimation of fall-related risks and incidents (*p* value < 0.0001, Figure 2B).

Another relevant aspect highlighted is the relationship between the availability of informational materials and the willingness of healthcare professionals to report patient falls. A lack of distributed brochures or written guidelines was significantly associated with increased uncertainty and reluctance to report fall events (*p* < 0.0001; Figure 2C).

### 5. Training and education needs

**Figure 3.**
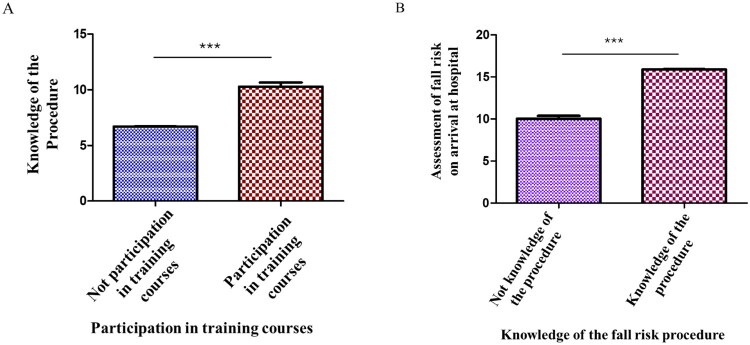
A) The histogram shows the correlation between the lack of participation in training courses and seminars on fall prevention and the limited knowledge of current institutional procedures; B) the histogram shows the statistical correlation between the assessment of the risk of falling when the patient arrives at the hospital and the knowledge of the fall risk procedure.

Outdated Training: 64.1% of nurses reported never having attended a training course on fall prevention. The data show a strong correlation between the lack of participation in training courses and seminars on fall prevention and the limited knowledge of current institutional procedures, particularly the guideline titled “Prevention and Hospital Management of Patient Falls” (*x*^2^ = 20.3; *p* < 0.0001, Figure 3A).

This study found also that when staff are well-informed about the established protocol for fall prevention, they are significantly more likely to perform a timely and accurate risk assessment at the time of hospital admission (*p* value <0.0001, Figure 3B).

Knowledge Gap Between Trained and Untrained Nurses: 2.9% of respondents rated fall prevention training as minimally useful (1–2 on a scale of 5). Training on fall prevention was significantly associated with improved adherence to safety practices and reduced fall-related risks (*p* = 0.0005).

### 6. Adherence to fall prevention protocols

Incomplete documentation: 47.7% indicated that the “Risk of Falls” informational brochure is not consistently available in their unit.

Lack of incident reporting: 56.9% of nurses and healthcare assistants declared they had never reported a patient fall to the Quality and Clinical Risk Office.

**Figure 4.**
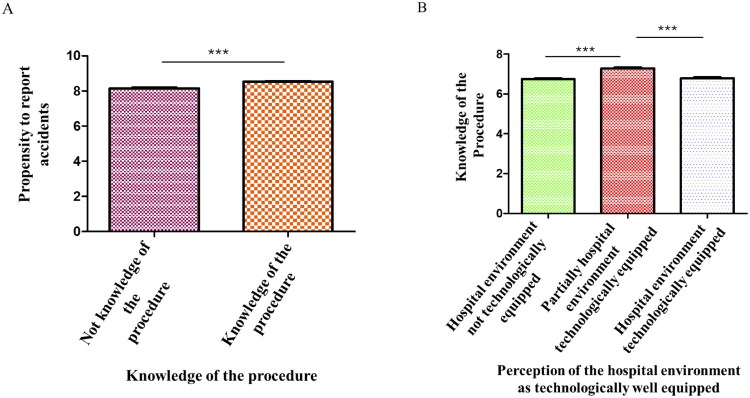
A) The histogram shows the significant correlation between knowledge of the procedure and the propensity to report accidents; B) the histogram shows the statistical correlation between the knowledge of the procedure by nurses and healthcare assistants and the positive or negative perception of the hospital environment as technologically well equipped to manage falls.

Correlation with procedure awareness: A statistically significant association was found between knowledge of the company procedure “Prevention and Hospital Management of Patient Falls” and the likelihood of reporting fall incidents (*p* < 0.0001; Figure 4A). Additionally, limited awareness of the procedure was significantly associated with reduced attention to fall risk assessment upon patient admission (*p* = 0.002). Respondents who reported inadequate focus during the initial evaluation phase also demonstrated greater difficulty in applying prevention guidelines correctly (*p* < 0.0001).

Another interesting aspect concerns the perception of risk, and the availability of technological requirements needed to manage falls.

In our study, knowledge of the fall prevention procedure was significantly associated with the perception of the hospital environment as being adequately equipped with technological resources for managing fall risk (p-value < 0.0001, Figure 4B).

## Discussion

In this study, we observed variability in fall prevention knowledge, participation in training programs, and perceived institutional support among nurses and healthcare assistants. Inconsistent prevention practices may have clinical and medico-legal implications, highlighting the value of clear protocols and structured staff training to support safe care environments [6,7].

Professional experience plays an important role in fall management. Previous studies have shown that nurses with over than 10 years of experience tend to exhibit greater competence in preventing falls, likely due to their extensive field exposure [8].

However, the findings of our study offer additional insights into the knowledge and attitudes of nurses and healthcare assistants regarding fall prevention. Despite the participants’ high level of clinical experience, some uncertainty remains regarding the interpretation and practical value of fall risk assessment tools [9]. Continuous professional education remains essential to address knowledge gaps and promote consistent, evidence-based approaches to fall prevention [8].

Nurses and healthcare assistants who participate in training courses or seminars on fall prevention are more likely to adopt effective practices in patient care, contributing to a significant reduction in the incidence of falls [10].

Knowledge of fall prevention procedures is closely linked to the propensity to report incidents, the perception of available resources, attention to fall risk assessment, and clinical experience in managing falls. These findings are consistent with previous research and underscore their critical role in enhancing patient safety practices [10,11].

Targeted education on safe mobilization techniques, accurate fall risk assessment, and appropriate use of assistive devices is particularly impactful in enhancing safety and promoting a proactive approach to fall prevention [12,13].

Therefore, experience combined with training can improve the management of falls. The interaction between these two factors is fundamental [13,14,15].

In line with the existing literature, our study explores the statistical correlation between professional experience (categorized as less than 5 years, less than 10 years, and more than 10 years) and the frequency of participation in training courses on fall prevention. This analysis offers valuable insights into how professional seniority influences engagement in educational initiatives and contributes to effective fall management strategies within the hospital setting.

In this study, the widespread support for environmental and technological interventions, such as bed and chair alarms, underscores the perceived utility of these tools in structured fall prevention programs. However, the concerns raised by some nurses and healthcare assistants regarding alarm overuse and the potential for patient distress reflect growing awareness of the limited evidence supporting their effectiveness [1,2]. These perspectives, aligned with recent international guidelines, highlight the need for a more nuanced and evidence-based approach that prioritizes individualized care and avoids reliance on interventions of questionable benefit [1,2].

Furthermore, effective interprofessional communication emerged as a key factor in improving adherence to fall prevention protocols. The high percentage of nurses emphasizing collaboration with healthcare assistants suggests that multidisciplinary teamwork plays a crucial role in maintaining safety standards. Institutions should consider reinforcing communication strategies through targeted training programs and shared responsibility models [15,16].

By contrast, the divergence in opinions regarding the use of standardized fall prevention protocols versus institution-specific guidelines reflects a lack of consensus within the healthcare community. While standardized tools promote uniformity and comparability across settings, customized protocols may offer greater adaptability to the unique operational and patient care needs of individual hospitals [15,16]. Future research should investigate the comparative effectiveness of these approaches in improving patient outcomes to better inform evidence-based policy and practice.

The overwhelming majority of respondents (∼95%) agree that maintaining a safe environment plays a crucial role in preventing falls, while a small minority disagree, indicating potential gaps in training or different perspectives on fall preventability.

To ensure a safe environment, healthcare professionals and nurses must develop greater awareness of fall risks, receive thorough training, and be familiar with the procedures currently in place within our institution, such as the protocol “Prevention and Hospital Management of Patient Falls.” Enhanced safety and awareness can only be achieved by continuously promoting learning and improvement of these procedures [16,17].

Another key factor is fostering a workplace culture in which nurses and other healthcare workers do not fear retaliation for reporting an incident. The adoption of reporting systems that enable fair assessment of root causes is essential for promoting transparency and driving quality improvement [17,18].

As an initial intervention, it is crucial that fall risk assessments for each hospitalized patient are regularly discussed during handovers. If this topic is not systematically addressed, there is a risk of failing to identify at-risk patients promptly, thereby compromising their safety and further diminishing their quality of life [1,18].

This improved adherence to protocols is directly associated with a measurable reduction in fall-related events, confirming the central role of ongoing professional education in promoting patient safety.

However, knowledge of procedures must also be accompanied by patient education, when possible. Actively involving patients, raising staff awareness, and correcting entrenched improper behaviors have proven to be key factors in fall prevention. Effective falls prevention requires indeed interdisciplinary collaboration, engaging a broad spectrum of stakeholders including physicians, physiotherapists, pharmacists, nurses, patients, and their families [1].

Knowledge is also reinforced by the availability of educational materials. The ability to easily access such resources appears to positively influence the reporting behavior of nurses and healthcare assistants. Indeed, the presence of brochures and clearly written guidelines enhances staff awareness and engagement in incident reporting. Accessible, well-structured information not only facilitates the correct execution of reporting procedures but also reinforces the overall culture of safety within the clinical setting [1,19,20].

### Limitations of the study

Several important limitations must be acknowledged in interpreting the results of this study. First, the study was conducted within a single Local Health Authority and while multiple hospital departments were involved, the results may not be fully generalizable to other institutions or healthcare systems with different organizational structures or resources.

Second, a major limitation concerns the lack of detailed and systematic data on actual fall events within the hospital. The absence of precise, up-to-date records on the frequency, circumstances, and risk factors associated with patient falls hinders the ability to objectively assess the effectiveness of the preventive measures currently in place. Furthermore, this gap prevents a longitudinal analysis of trends over time, limiting the opportunity to conduct robust comparative evaluations.

Third, although the questionnaire provided valuable insights into perceived knowledge and attitudes, the study relied on self-reported data, which may be subject to social desirability bias or inaccuracies in recall. Objective measures of actual clinical practice or fall event documentation were not included.

Finally, while the sample size (*n* = 295) was adequate for preliminary analysis, a larger and more geographically diverse population would increase the external validity of the findings. Future studies should aim to expand the sample, incorporate longitudinal follow-up, and integrate hospital incident data to enable a more comprehensive and evidence-based evaluation of fall prevention strategies.

## Conclusions

This study reinforces the critical role of healthcare professionals in fall prevention and underscores the need for comprehensive, multifactorial strategies to achieve meaningful outcomes. Effective fall prevention requires the integration of multiple approaches, including continuous staff training, environmental modifications, rehabilitation support, timely response to patient needs, the use of appropriate assistive devices, and personalized interventions tailored to individual risk profiles.

There is no single, definitive solution to preventing falls in hospital settings. However, education consistently emerges as the most reliable and impactful intervention. The most effective outcomes are achieved through a coordinated strategy that combines technological resources, clinical expertise, a safe and supportive care environment, and the active engagement of all members of the healthcare team.

Overall, these findings emphasize the importance of sustained educational efforts, continuous policy refinement, and strong interdisciplinary collaboration to strengthen fall prevention practices. Future research should focus on evaluating the effectiveness of targeted, context-specific interventions in improving adherence to best practices and reducing the incidence of falls in clinical settings.

## Supplementary Material

survey.pdf

## Data Availability

Full anonymous data from the survey are available on request.
