# Supplementary material for: Perceived knowledge and attitudes toward fall prevention among nurses and healthcare assistants: a Cross-Sectional survey study
Source: Ann Med. 2025 Sep 19;57(1):2559127. doi: 10.1080/07853890.2025.2559127 (PMC12451957; doi:10.1080/07853890.2025.2559127)
Supplement: survey.pdf [file IANN_A_2559127_SM0687.pdf]

Perceived Knowledge and Attitudes Toward Fall Prevention Among Nurses and Healthcare Assistants at ASL02 Lanciano-Vasto-Chieti.

*Indicates a required question

Socio-demographic and professional information.

1. Age: *

   Only one

   - From 22 to 25 years
   - From 26 to 35 years
   - From 36 to 45 years
   - From 46 to 55 years
   - Over 55 years

2. Gender: *

   Only one

   - Male
   - Female

3. Profession: *

Only one

- Professional nurse
- Healthcare assistant

4. Education level *

- Bachelor's Degree in Nursing
- Master's Degree in Nursing and Midwifery Sciences
- First-Level University Master
- Second-Level University Master
- PhD (Doctorate of Research)
- Basic Healthcare assistant Course
- Specialized Healthcare assistant Course
- Other

5. Years of work experience: *

Only one

- < 5 years
- Up to 10 years
- > 10 years

6. Hospital facility where you currently work: *

___

7. Healthcare area of affiliation: *

Only one

- Medical Area
- Surgical Area
- Maternal and Child Area
- Emergency and Admission Area

- Mental Health Area
- Service Area
- Cardiology Area
- Community/Outpatient Area

8. Unit/Department of affiliation: *

____

9. Years of experience within your current Unit: *

Only one

- < 5 years
- Up to 10 years
- > 10 years

10. Weekly working hours:*

Only one

- Shift work in the Unit
- Daytime schedule in the Unit
- Daytime schedule in the Outpatient Clinic
- Other

11. Are you aware of the issue related to patient falls in the hospital?

Only one

- Yes
- No
- I do not consider it a problem

12. Have you ever attended training courses and/or seminars on patient falls in the hospital? *

Only one

- Yes
- No

13. If you answered YES to the previous question, please specify how many training courses and/or seminars you have attended.

Only one

- < 5 training courses or seminars
- > 5 training courses or seminars

14. When did you receive specific training on patient falls in the hospital? (seminars/courses/workshops)

Only one

- < 2013
- > 2015
- Never

15. Would you be interested in attending training courses that address the issue of patient falls in the hospital? *

Only one

- Yes
- No

16. How useful do you consider specific training for healthcare workers on fall prevention in the hospital setting? (1 represents the lowest and 5 the highest score) *

Only one

1	2	3	4	5
○	○	○	○	○

17. Are you familiar with the current procedure in our Healthcare Facility ? *

Only one

- Yes
- No

18. If you answered YES to the previous question, how adequate do you consider it for the type of patients you care for? (1 represents the lowest score and 5 the highest) *

Only one

	1	2	3	4	5
	◯	◯	◯	◯	◯

19. Have you ever reported or contributed to reporting an accidental patient fall to the Quality and Clinical Risk Office? *

Only one

- Yes
- No

20. If you answered Yes to the previous question, how difficult did you find the reporting procedure? (1 represents the lowest level of difficulty and 5 the highest)

Only one

	1	2	3	4	5
	◯	◯	◯	◯	◯

21. If you were in the position of having to report an accidental fall event, would you feel uncomfortable and/or hesitant in doing so? *

Only one

- Yes
- No

22. Do you believe that the nursing staff and healthcare assistants in your Unit pay sufficient attention to fall risk assessment at the time of patient admission? * *Only one*

- Yes
- No

23. Is the informational brochure on “Fall Risk” for patients and families always available for distribution in your Unit?*

Only one

- Yes
- No

24. During shift handover, is the fall risk assessment of hospitalized patients addressed? *

Only one

- Yes
- No
- Partially

25. Have you ever had direct experience in managing falls in frail older patients? *

Only one

- Yes
- No

26. Have you ever had direct experience in managing falls in frail older patients? *

Only one

- Yes
- No

27. Nurses and healthcare assistants who take charge of patient care tend to be more effective than relying solely on a standardized assessment scale for identifying patients at risk of falling. *

Only one

- True
- False

28. Select the correct sequence of the three-step fall prevention process from the options below. *

Only one

- 1 Proper management and completion of documentation regarding fall prevention in the hospital, 2 Fall risk screening, 3 Development of a personalized fall prevention plan.
- 1 Fall risk screening, 2 Proper management and completion of documentation regarding fall prevention in the hospital, 3 Development of a personalized fall prevention plan.
- 1 Fall risk screening, 2 Development of a personalized fall prevention plan, 3 Proper management and completion of documentation regarding fall prevention in the hospital.

29. A 75-year-old male patient with a history of recent falls and osteoporosis is admitted for severe abdominal pain. Would the risk of injury be higher if the patient were to fall at his age? *

Only one

- True
- False

30. One of the reasons why some hospitalized patients fall is that their fall prevention plan is not followed. *

Only one

- True
- False

31. Can patient falls be prevented by providing them, as much as possible, with a safe environment? (For example: a well-lit path to the bathroom, a clutter-free room, proper footwear) *

Only one

- True
- False

32. “Involvement” in fall prevention means that the nurse (also with the support of the healthcare assistant) completes the fall risk assessment and the prevention plan, while educating the patient on the main risk factors. *

Only one

- True
- False

33. All hospitals are different from one another; therefore, a separate fall risk assessment form should be developed for each facility. *

Only one

- True
- False

34. Fall risk assessment scales identify patients who are more likely to fall because they present with one or more physiological problems. *

Only one

- True
- False

35. In your opinion, when there is effective communication between nurses and healthcare assistants, is the likelihood of patients following the fall prevention plan increased? *

Only one

- True
- False

36. Patients classified as low risk for falls do not require a fall prevention assessment. *

Only one

- True
- False

37. If available in our Healthcare Facility, do you think bed and/or chair alarms should be activated for all patients identified as high risk for falls? *

Only one

- True
- False

Created with Google Moduli
